# TSPphg Lysin from the Extremophilic *Thermus* Bacteriophage TSP4 as a Potential Antimicrobial Agent against Both Gram-Negative and Gram-Positive Pathogenic Bacteria

**DOI:** 10.3390/v12020192

**Published:** 2020-02-09

**Authors:** Feng Wang, Xinyu Ji, Qiupeng Li, Guanling Zhang, Jiani Peng, Jun Hai, Yao Zhang, Baiquan Ci, Hongwei Li, Yan Xiong, Xianyu Deng, Lianbing Lin

**Affiliations:** 1 Faculty of Life Science and Technology, Kunming University of Science and Technology, 727 South Jingming Road, Kunming 650500, China; wangfeng076@hotmail.com (F.W.);; 2 Engineering Research Center for Replacement Technology of Feed Antibiotics of Yunnan College, 727 South Jingming Road, Kunming 650500, China

**Keywords:** bacteriophage, endolysin, thermostable lytic protein, antibiotic-resistant bacteria, antimicrobial drug, *Staphylococcus aureus*, skin infection

## Abstract

New strategies against antibiotic-resistant bacterial pathogens are urgently needed but are not within reach. Here, we present in vitro and in vivo antimicrobial activity of TSPphg, a novel phage lysin identified from extremophilic *Thermus* phage TSP4 by sequencing its whole genome. By breaking down the bacterial cells, TSPphg is able to cause bacteria destruction and has shown bactericidal activity against both Gram-negative and Gram-positive pathogenic bacteria, especially antibiotic-resistant strains of *Klebsiella pneumoniae*, in which the complete elimination and highest reduction in bacterial counts by greater than 6 logs were observed upon 50 μg/mL TSPphg treatment at 37 °C for 1 h. A murine skin infection model further confirmed the in vivo efficacy of TSPphg in removing a highly dangerous and multidrug-resistant *Staphylococcus aureus* from skin damage and in accelerating wound closure. Together, our findings may offer a therapeutic alternative to help fight bacterial infections in the current age of mounting antibiotic resistance, and to shed light on bacteriophage-based strategies to develop novel anti-infectives.

## 1. Introduction

Currently, bacterial resistance has been reported in all WHO regions and identified for every antimicrobial drug developed to date [1,2]. In particular, the recent increase in bacterial strains showing resistance to almost all classes of antibiotics commonly used in human medicine has become a serious threat to public health worldwide [3,4]. Therefore, there is an urgent need to develop alternatives to conventional antibiotics for use in the treatment of infectious diseases.

Bacteriophages (also termed phages) have been found to be natural agents fighting against bacterial infections in the early part of the 20th century, and they have showed plenty of advantages in controlling infections in a wide range of experimental animal models such as chickens and cattle [5,6,7]. In fact, the emergence of a phage technology revolution is not just limited to whole phages. Recently, their lytic enzymes (also known as lysins), which can degrade peptidoglycan and thus digest a host’s bacterial cell wall, have also gained new ground as promising antibacterial agents due to their safety, broad substrate spectrum and low possibility of developing resistance [8,9,10]. When applied exogenously in the treatment of bacterial infections, recombinant phage lysins can exhibit similarly effective antibacterial performance to their native counterparts, supporting the wide application of these agents in the fields of biotechnology, medicine, food and agriculture [11,12]. Interestingly, lysins can also evolve with time based on the needs of phages to release from their infected hosts [13].

In the present study, we sequenced the whole genome of an extremophilic *Thermus* phage TSP4 that was isolated from Tengchong hot spring in Yunnan Province of China at a temperature of 70 °C with a pH of 7.0 [14], which is particularly interesting because of the thermostability of its lytic proteins and nucleotide metabolism system. In the process, we identified a novel phage lysin named TSPphg and assessed its in vitro and in vivo antibacterial activity against a panel of antibiotic-resistant strains.

## 2. Materials and Methods

### 2.1. Ethics Statement

The experimental mice (Kunming mice) were purchased from the Animal Center of Kunming Medical University (Kunming, China). All procedures were conducted in accordance with the Regulations for the Administration of Affairs Concerning Experimental Animals, and approved by the Animal Research Ethics Committee of Faculty of Life Science and Technology, Kunming University of Science and technology on 15 May 2019 (document no. 2019-LBL-001).

### 2.2. Bacterial Strains and Culture Conditions

Strains of *Escherichia coli, Salmonella paratyphi* B and *Bacillus subtilis* were cultivated at 37 °C with shaking (150 rpm) in Luria Broth (LB) medium (10 g/L tryptone, 10 g/L yeast extract, 5 g/L NaCl, pH = 7.2 ± 0.2). *Staphylococcus epidermidis*, *Micrococcus luteus* and *Staphylococcus aureus* strains were grown with shaking at 150 rpm in Nutrient Agar medium (10 g/L tryptone, 3 g/L beef extract, 5 g/L NaCl, pH = 7.2 ± 0.2) at 37 °C, and clinical isolates of *Klebsiella pneumoniae* were cultured at 37 °C in Brain Heart Infusion (BHI) medium (10 g/L tryptone, 12.5 g/L brain infusion powder, 5 g/L beef heart powder, 5 g/L NaCl, 2 g/L glucose, 2.5 g/L Na_2_HPO_4_, pH = 7.4 ± 0.2) with shaking at 150 rpm. *Thermus* sp. TC4 that belongs to the genus *Thermus* was grown at 60 °C on DSM88 medium with shaking as previously reported [14]. The bacteria used as substrates for TSPphg were purchased from the American Type Culture Collection (*S. aureus* ATCC6538, *S. epidermidis* ATCC12228 and *M. luteus* ATCC4698) or National Center for Medical Culture Collections (*S. paratyphi* B CMCC(B)50094 and *B. subtilis* CMCC(B)63501). The nine different isolates of *K. pneumoniae* (see Table 1), a multidrug-resistant *S. aureus* strain (1606BL1486) and *E. coli* O157 (KUST401) were kindly provided by Prof. Xueshan Xia and Prof. Yuzhu Song in the Research Center of Molecular Medicine of Yunnan Province, Kunming University of Science and Technology, with their antibiotic resistance patterns determined based on susceptibility tests according to the CLSI (Clinical and Laboratory Standards Institute) guidelines. All other bacteria were stored in our laboratory. *E. coli* strains DH5a and BL21 were used for plasmid construction and recombinant protein overproduction, respectively. When necessary, the media were supplemented with ampicillin at 100 μg/mL or kanamycin at 50 μg/mL. All strains were stored at −80 °C.

### 2.3. Genome Sequencing and Annotation

The genomic DNA of phage TSP4 was extracted and purified by a DNA extraction kit (Omega Bio-tek Inc., Norcross, GA, USA) and sequenced in shanghai majorbio company by Sanger sequencing. Open reading frames (ORFs) were predicted with ORF finder on the NCBI website (https://www.ncbi.nlm.nih.gov/orffinder). All ORF predictions were refined by blasting ORFs to the NCBI database. The conserved domain analysis was carried out based on the NCBI Conserved Domain Database (CDD v3.17, last update: 2019-04-03). This genome project has been deposited in GenBank under the accession number MH992131.1.

### 2.4. Production and Purification of Recombinant Protein TSPphg

The highly efficient expression of TSPphg in the host *E. coli* BL21 cells and its rapid purification using the special thermolysis method were already described in detail in our previous publication [15]. Briefly, *TSPphg* gene was amplified by PCR with gene-specific primers from the phage TSP4 genome (forward: 5’-CATGCCATGGCAATGCGTCTACCGACTAAGAC-3’ and reverse: 5’-CCGCTCGAGTTTACCTCCTAGCAACTTGG-3’). The 5′ ends of forward and reverse primers contained NcoI and XhoI restriction sites (underlined), respectively. The modified primers were used to amplify the *TSPphg* gene for directional cloning into the expression vector pET-28a. The PCR program was performed as follows: initial denaturation at 94 °C for 3 min, followed by 30 cycles of 94 °C for 45 s, 58 °C for 30 s, and 72 °C for 90 s. *E. coli* BL21 cells harboring the pET-28a-*TSPphg* vector were then used as the host for recombinant protein expression. Lactose (1 g/L) was used for induction to overproduce the phage lysin TSPphg. After induction, cell pellets were collected by centrifuging at 12,000 g for 10 min at 4 °C, then resuspended in phosphate-buffered saline (PBS) containing 137 mM NaCl, 2.7 mM KCl, 4.3 mM Na_2_HPO_4_, and 1.4 mM KH_2_PO_4_ with a pH of 7.4. The recovery of thermostable TSPphg protein was performed by a direct heat treatment at 55 °C for 30 min to precipitate unstable host proteins. Subsequently, the samples were centrifuged at 12,000× *g* for 10 min to pellet debris, and filtered using 0.22-μm pore-size filters (Sartorius, Ulm, Germany). The final purified TSPphg dissolved in PBS was confirmed by 12% sodium dodecyl sulfate polyacrylamide gel electrophoresis (SDS-PAGE). Finally, from 1 L of the host *E. coli* BL21 culture we could obtain approximately 79 mg of TSPphg.

### 2.5. Assessing Thermostability of TSPphg and Effects of pH, NaCl and EDTA on Its Activity

To evaluate the characteristics of TSPphg, the host bacterium for phage TSP4, *Thermus* sp. TC4 (GenBank accession: GQ853550.1) was used as the substrate and cultured in DSM88 medium at 60 °C until an OD_600_ of 0.4-0.5 was reached as previously described [14]. Then, the bacterial cells were collected by centrifuging at 1000× *g* for 10 min at 4 °C, washed twice, then resuspended in PBS (137 mM NaCl, 2.7 mM KCl, 4.3 mM Na_2_HPO_4_, 1.4 mM KH_2_PO_4_, pH 7.4). For thermostability assays, TSPphg at 50 μg/mL prepared in PBS was first heated at different temperatures (from 10 to 85 °C) for 30 min; then, its lytic activity was measured by the standard turbidity reduction assay against 10^8^ of *Thermus* sp. TC4 cells at 60 °C in a standard 96-well microtiter plate (ShenYing Biotechnology, Haimeng, China). The lytic activity of TSPphg was determined by a decrease in OD_600_ in a plate reader (Wisdom Applied Science, mode 6500, Newark, DE, USA), and all plates were shaken for 4 s before each measurement. To study the effects of different pH values and NaCl concentrations on the activity of TSPphg, exponential phases of *Thermus* sp. TC4 cells (OD_600_ = 0.4–0.5) were exposed to 50 μg/mL of TSPphg at 60 °C for 1 h over a range of pH values (4 to 10) or NaCl concentrations (from 0 to 1000 mM) [16]. Lysis of TC4 cells by TSPphg was also monitored in the absence (non-treated control) or in the presence of ethylenediaminetetraacetic acid (EDTA, from 0.5 to 5 mM). For all experiments, control wells were run in parallel, and the lytic activity of TSPphg was calculated at specific condition as follows: (OD_600_ (buffer only) − OD_600_ sample (lysin added))/initial OD_600_, as previously described [17,18]. All assays were repeated in triplicate.

### 2.6. In Vitro Antibacterial Activity Assays

To examine the antibacterial activity of TSPphg, various Gram-negative or Gram-positive bacteria were grown and tested as described previously [19]. Briefly, the bacteria were grown at 37 °C in a rotary shaker at 150 rpm until an OD_600_ of 0.6–0.8 was reached, then centrifuged at 1000 g for 10 min at 4 °C, washed twice, and resuspended in PBS containing 137 mM NaCl, 2.7 mM KCl, 4.3 mM Na_2_HPO_4_, and 1.4 mM KH_2_PO_4_ at a pH of 7.4. For time-kill curve experiments, approximately 10^7^ CFU/mL of bacterial cells were treated with increasing concentrations of TSPphg (17, 34, and 68 μg/mL) in PBS (pH 7.4) that was chosen based on an analysis of the effects of different pH values and NaCl concentrations on the activity of TSPphg. After incubation at 37 °C and 150 rpm for 0 to 90 min (samples were taken at an interval of 10 min), aliquots were serially diluted and spread onto LB agar plates. Subsequently, viable cells and log kills at different time points were evaluated after overnight incubation at 37 °C. The experiments were repeated thrice. To assess the antibacterial spectrum of TSPphg (see Table 1), a panel of Gram-negative and Gram-positive bacteria (from 10^5^ to 10^8^ CFU/mL) were mixed with 50 μg of TSPphg dissolved in PBS to make a final volume of 1 mL and a final concentration of 2.6 µM. The mixtures were then incubated at 37 °C for 1 h, and the viable cell numbers were counted by plating on agar plates. The antimicrobial activity of TSPphg was calculated as a relative decrease in logarithmic units after the indicated time points as follows: Log_10_ (N_0_/N_i_), N_0_ = number of untreated cells (in the control) and N_i_ = number of cells counted after treatment [17,18]. All assays were performed with at least three biological replicates. The limit of detection for bacterial counts in agar plate was 10 colony-forming units (CFU)/mL in this study.

### 2.7. Mouse Model of Skin Damage and TSPphg Treatment

A mouse model of skin damage was established using the female Kunming mice according to the methods described in previous studies [20,21]. Briefly, the hair on an approx. 2 cm^2^ section of the dorsum of each mouse was shaved and cleansed with 70% alcohol, then the epidermis was stripped using autoclave tape to induce skin damage. The damaged skin was topically colonized with the multidrug-resistant *S. aureus* (strain No. 1606BL1486) by using 20 μL (1 × 10^5^ CFU) cultures suspended in PBS. All mice were housed individually to avoid cross-infection and traumatic damage to the wounds by other animals. After 24 h of colonization, 100 μL of 50 μg/mL TSPphg dissolved in PBS or 100 μL of 50 μg/mL kanamycin were applied to the wounds, with 100 μL PBS as the negative control. All mice were treated once a day with TSPphg, kanamycin or PBS for seven days, and the wounds were photographed after treatment at different time points (*n* = 4 mice per group per experiment). Subsequently, the percentage of wound healing was calculated, and the bacterial loads in skin wounds were evaluated by serial dilution and plating on LB agar plates [22].

### 2.8. Scanning Electron Microscope (SEM) Analysis

The effects of exogenous TSPphg treatment on bacterial cell lysis were analyzed using a scanning electron microscope (Quanta 200, FEI, Holland) as previously described [22]. In brief, the bacteria were grown at 37 °C until an OD_600_ of 0.6–0.8 was reached, then they were collected by centrifugation at 1000 g for 5 min at 4 °C and washed twice with PBS; finally, 10^5^ CFU/mL of bacterial cells were incubated with 50 μg/mL TSPphg and dissolved in PBS at 37 °C for 1 h. Subsequently, the bacterial lysates were fixed with 2.5% glutaraldehyde at 4 °C for 6 h and dehydrated in a graded ethanol series (30%, 50%, 60%, 70%, 80%, and 90%) for 20 min each time. The samples were further dried at room temperature for 24 h, then used for SEM analysis according to the manufacturer’s instructions.

### 2.9. Statistical Analysis

Statistical differences of quantitative data were determined by a Student’s *t*-test (between two groups) or a one-way analysis of variance (ANOVA, among three or more groups) followed by a Bonferroni correction using the R software version 3.2.2. All data were expressed as mean ± standard deviation (SD) of *n* = 3 independent replicates. The two-tailed *p* value < 0.05 was considered statistically significant.

### 2.10. Nucleotide and Protein Sequence Accession Number

The complete genome sequence of *Thermus* phage TSP4 and protein sequence of TSPphg lysin were deposited at GenBank under the accession numbers MH992131.1 and QAY18185.1, respectively.

## 3. Results

### 3.1. Identification of TSPphg by Sequencing and Annotation of Phage TSP4 Genome

**Figure 1:**
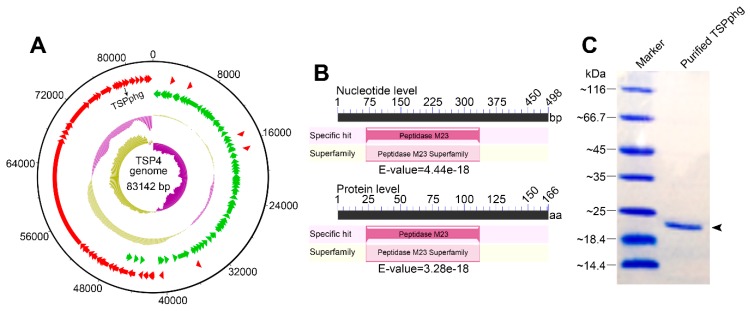
Identification of TSPphg by sequencing the complete genome sequence of *Thermus* phage TSP4. (**A**) Genome map of TSP4, from inside to outside: circle 1 displays the GC skew ((G − C)/(G + C)); circle 2 shows the GC percentage plot; circles 3 and 4 denote the ORFs on the minus (‘−’) strand (green) and plus (‘+’) strand (red), respectively; circle 5 demonstrates the numbered scale with an interval of 4 kb. (**B**) The conserved domain analysis of TSPphg at both nucleotide and protein levels. (**C**) SDS-PAGE analysis of the purified TSPphg in this study. The black arrowhead denotes the band of recombinant TSPphg lysin.

Previously, we isolated and characterized a *Thermus* phage named TSP4 from the Tengchong hot spring in China, and the host bacterium for phage TSP4 was *Thermus* sp. TC4 (GenBank accession: GQ853550.1), which was found to be a Gram-negative, rod-shaped, aerobic thermophile thriving at 40–80 °C [14]. In this study, we further sequenced the complete genome of phage TSP4. As shown in Figure 1A, it comprises one circular chromosome of 83,142 bp with a 56% GC content. Bioinformatic analysis predicted 108 protein-coding genes but no rRNA or tRNA genes within the genome of TSP4 (GenBank accession number MH992131; Table S1). Among them, TSPphg was predicted to be a putative phage lysin containing a conserved M23 peptidase domain (NCBI domain architecture ID: 10480195). It is thought to be highly similar to the M23 peptidase family (accession ID: pfam01551), which comprises specific hydrolytic activity on bacterial peptidoglycans, and thus could result in lysis of bacterial cells (Figure 1B). The natural DNA sequence of the *TSPphg* gene in the phage TSP4 genome was cloned by PCR. Overexpression and rapid purification of heat-stable TSPphg protein from the host *E. coli* BL21 cells were previously described in our publication [15], and there is no difference in the sequence between recombinant and wild-type TSPphg lysin. The purified TSPphg lysin used in this study was confirmed by 12% SDS-PAGE (Figure 1C).

Next, the effects of pH and NaCl on the lytic activity of TSPphg against the host strain for phage TSP4, *Thermus* sp. TC4, were examined. The pH range for TSPphg was measured from pH 4.0 to 10.0 and the highest activity was observed at pH 8.0 (Figure S1A). Based on this data, further examination of NaCl concentration dependence was performed at pH 8.0. As shown in Figure S1B, we found that TSPphg displayed optimal activity at 100 mM NaCl, making it more attractive for applications in physiological conditions where the NaCl concentration is 130 mM [17,23]. Unexpectedly, the addition of EDTA did not have enhanced effect on the activity of TSPphg (Figure S2), which is in line with PlyE146, a phage lysin comprising a highly positively charged C-terminal domain [24]. Based on these results, the common phosphate-buffered saline (PBS), which is a balanced salt solution with similar physiological conditions, was used as the working buffer for TSPphg for the following antibacterial assays, unless otherwise stated. Moreover, the thermostability of TSPphg was tested in a range of temperatures from 10 to 85 °C. As shown in Figure S3, TSPphg maintained more than 90% activity after a 30 min heat treatment between 40 and 70 °C, suggesting its resistance to denaturation at high temperatures.

### 3.2. Bactericidal Activity of TSPphg against both Gram-negative and Gram-positive Bacteria

**Figure 2:**
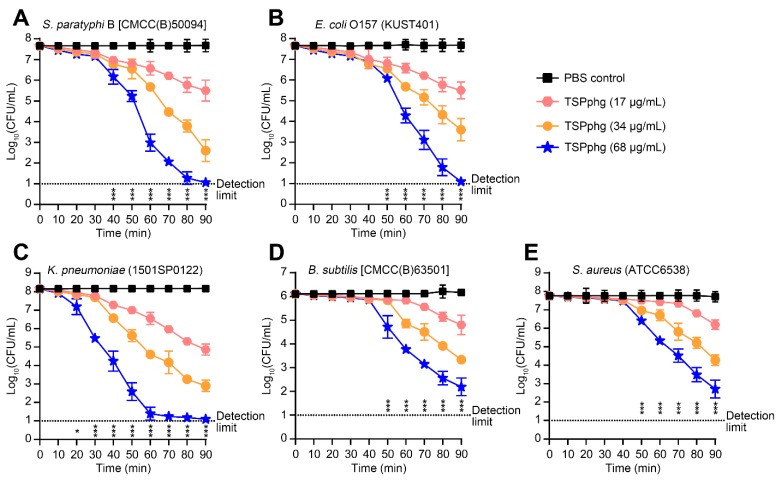
Time-kill assays. Antimicrobial activity of TSPphg against *Salmonella paratyphi* B (**A**), *Escherichia coli* O157 (**B**), *Klebsiella pneumoniae* (**C**), *Bacillus subtilis* (**D**) and *Staphylococcus aureus* (**E**). *S. paratyphi* B, *E. coli* O157 and *K. pneumoniae* are Gram-negative bacteria while *B. subtilis* and *S. aureus* are Gram-positive bacteria. Bacterial cell numbers were counted as Log_10_ (CFU/mL) at different time points as denoted. CFU means colony-forming unit. Data were presented as mean ± standard deviation (*n* = 3) and were analyzed using one-way analysis of variance (ANOVA) with a Bonferroni correction. *, *p* < 0.05; ***, *p* < 0.001. The dotted line shows the limit of detection (<10 CFU/mL).

Further, we carried out dose-dependent experiments and measured the antimicrobial activity of TSPphg against both Gram-negative (*S. paratyphi* B, *E. coli* O157 and *K. pneumoniae*) and Gram-positive (*B. subtilis* and *S. aureus*) bacteria at 37 °C in vitro. It can be seen from Figure 2 that the bactericidal activity of the TSPphg increased proportionally to its concentration, and it showed antimicrobial effects on both Gram-negative (Figure 2A–C) and Gram-positive bacterial strains (Figure 2D,E). It is noteworthy that with the concentration of 68 μg/mL, incubation of TSPphg with different bacterial cell suspensions for 1.5 h can result in significant reduction in the bacterial counts by 6.61 ± 0.19, 6.57 ± 0.19, 7.07 ± 0.01, 3.92 ± 0.37, and 5.06 ± 0.48 logs for *S. paratyphi* B, *E. coli* O157, *K. pneumoniae*, *B. subtilis* and *S. aureus*, respectively (*p* = 4.49E-07, 4.94E-07, 3.81E-12, 5.12E-05 and 5.27E-05 for TSPphg vs the PBS-treated control in the strains of *S. paratyphi* B, *E. coli* O157, *K. pneumoniae*, *B. subtilis* and *S. aureus*, respectively). Overall, these results suggest the potential of TSPphg for antimicrobial applications in biotechnology.

### 3.3. Antibacterial Activity of TSPphg against Antibiotic-Resistant Strains of Klebsiella Pneumoniae

It is well known that *K. pneumoniae* is a common cause of severe infections including pneumonia, endophthalmitis and meningitis [25]. Because of its hypervirulence and general occurrence of resistance to most antibiotics, in recent years infections caused by *K. pneumoniae* have become progressively difficult to treat [26,27]. In this study, we examined the antibacterial activity of TSPphg against nine different antibiotic-resistant strains of *K. pneumoniae* that are exceedingly hard to deal with in clinic practice. As shown in Table 1, TSPphg exhibited bactericidal activity against all these strains with a log reduction in bacterial counts between 1 and >6 logs. Particularly, we even observed the complete eradication (no bacteria colony was observed in agar plate after treatment) of *K. pneumoniae* strains 13A14918, 14A0287, and 1412SP0200 (>6.48, >6.74, and >6.82 logs, respectively) upon exposure of these bacterial cells to TSPphg (50 μg/mL) at 37 °C for 1 h. Besides the antibacterial activity against these Gram-negative strains of *K. pneumoniae*, TSPphg also showed activity against several strains of Gram-positive bacteria (*B. subtilis*, *S. epidermidis*, *M. luteus*, and *S. aureus*). For example, as demonstrated in Table 1, considerable TSPphg lysin activity was observed in the case of a highly dangerous and multidrug-resistant Gram-positive *S. aureus* (strain No. 1606BL1486) by a log reduction of 2.96 ± 0.43.

Larpin et al. have reported that the in vitro bactericidal concentration of PlyE146, a novel phage lysin targeting Gram-negative bacteria, was 400 μg/mL (as defined by a ≥3 log_10_ decrease in cell counts compared to that in the absence of lysin) [24]. Accordingly, as shown in Figure S4, we found that the bactericidal concentration of TSPphg was approximately in a range of 30–80 μg/mL. Compared with recent studies [16,18,24,28], this concentration is at a relatively low level.

### 3.4. TSPphg Accelerates Wound Healing in a Murine Skin Infection Model

**Figure 3:**
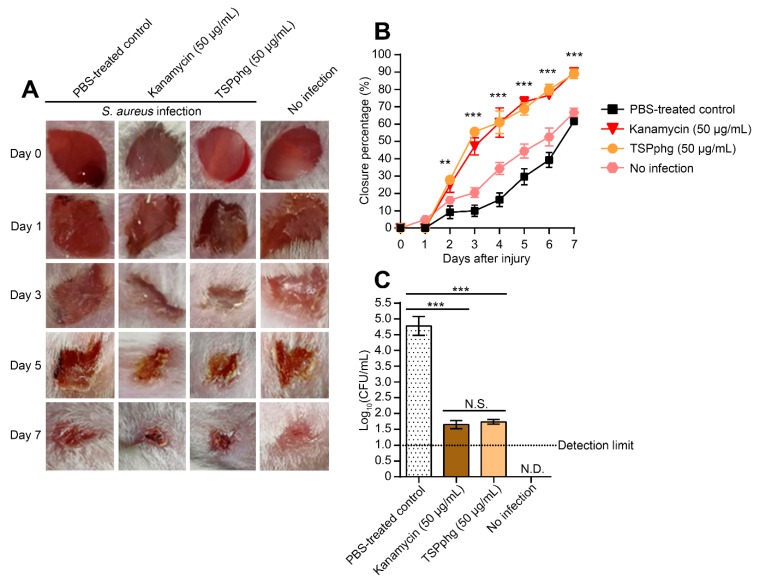
TSPphg promotes wound healing in a mouse model of a multidrug-resistant *S. aureus*-infected skin wound. (**A**) Photographs of the infected wound sites treated with 100 μL PBS, 100 μL of 50 μg/mL kanamycin, or 100 μL of 50 μg/mL TSPphg, and the group without infection. (**B**) Wound healing percentage in all groups. Data were presented as mean ± standard deviation (*n* = 4 mice). *p* values were calculated using one-way analysis of variance (ANOVA) with a Bonferroni correction. **, *p* < 0.01; ***, *p* < 0.001. (**C**) In vivo decolonization activity of TSPphg in experimental mice infected with multidrug-resistant *S. aureus* (1 × 10^5^ CFU/mouse). At 24 h after colonization, the mice were continuously treated once a day with 100 μL PBS, 100 μL of 50 μg/mL kanamycin or 100 μL of 50 μg/mL TSPphg. At day 7, the bacterial loads in the wounds were examined. Data were presented as mean ± standard deviation of *n* = 4 mice, and *p* values were determined using a Student’s *t*-test. ***, *p* < 0.001; N.D., non-detectable; N.S., not significant (*p* > 0.05).

To study the in vivo efficacy of TSPphg, we further established a mouse model of a 1606BL1486-infected skin wound. When the shaved areas of Kunming mice were topically colonized with 20-µL (1 × 10^5^ CFU) 1606BL1486 cultures, the skin wound became red and swollen. After 24 h of bacterial challenge, the mice were daily treated with 100 μL PBS, 100 μL of 50 μg/mL kanamycin, or 100 μL of 50 μg/mL TSPphg dissolved in PBS. As shown in Figure 3A,B, compared with groups of PBS-treated or no infection control, wound closure was considerably accelerated in both TSPphg and kanamycin treated groups. At day 7, the infected wound areas shrank by 89 ± 2.74% in the TSPphg group and 90 ± 1.26% in the kanamycin group; both were significantly higher than the PBS-treated group (*p* = 1.41E-04 and 2.73E-05 for TSPphg vs the PBS-treated control, and kanamycin vs the PBS-treated control, respectively). However, there was no significant difference between TSPphg and kanamycin treatment (*p* = 0.60). Moreover, we found that after TSPphg treatment, the bacterial colony counts successfully decreased from the starting 5 × 10^6^ to 55 ± 9 CFU/mL (*p* = 6.93E-05 for TSPphg vs the PBS-treated control, Figure 3C). These results suggest the in vivo efficacy of TSPphg in removing *S. aureus* from the skin damage and in accelerating wound closure, and warrant further exploration and optimization of its efficacy to develop TSPphg as a treatment option for drug-resistant bacterial infections in humans or poultry.

### 3.5. SEM Observations of the Bacteriolytic Activity of TSPphg

**Figure 4:**
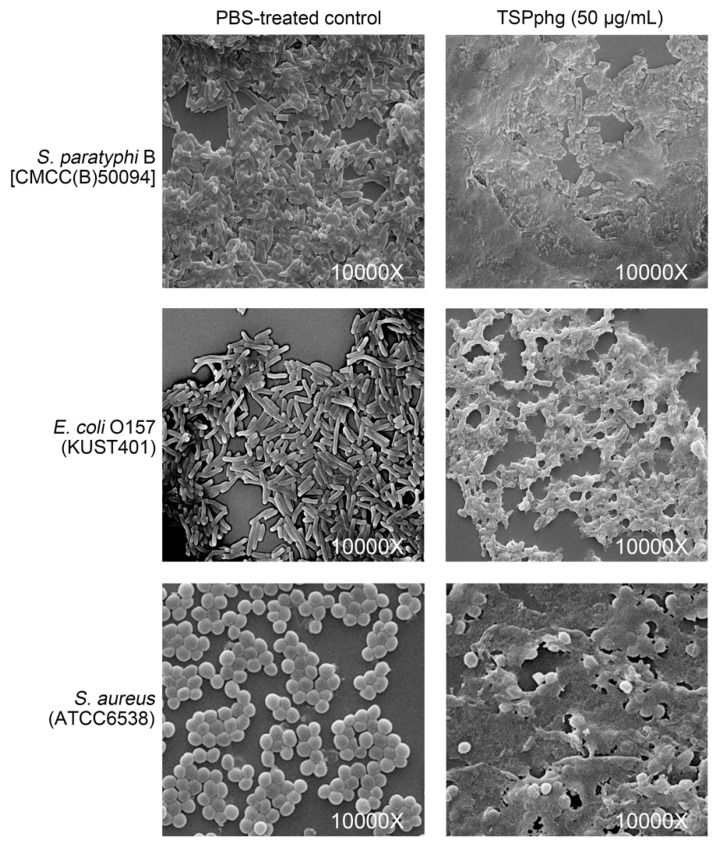
Scanning electron microscopy showing the effects of exogenous TSPphg treatment on the bacterial cell lysis of both Gram-negative (*S. paratyphi* B and *E. coli* O157) and Gram-positive (*S. aureus*) strains. For all tests, the 10^5^ CFU/mL of bacterial cells was treated with or without 50 μg/mL TSPphg dissolved in PBS at 37 °C for 1 h. The magnification is 10,000 times the original size.

To further investigate the detailed view of the bacterial cells after TSPphg treatment, scanning electron microscope (SEM) analysis was performed and the bacteriolytic activity of TSPphg against *S. paratyphi* B (CMCC(B)50094), *E. coli* O157 (KUST401), and *S. aureus* (ATCC6538) were tested. As shown in Figure 4, SEM of these strains upon exposure to 50 μg/mL TSPphg at 37 °C for 1 h exhibited disrupted cells with extruding materials and cell debris, suggesting that exogenous TSPphg treatment could cause the destruction of bacteria and thus release of their intracellular components. These findings are consistent with the above-mentioned in vitro and in vivo antimicrobial activity of TSPphg, and further confirm its function as a phage lysin and thus as a promising phage-derived antimicrobial agent.

## 4. Discussion

The rapid increase in prevalence of antibiotic-resistant strains has encouraged investigations into the role of bacteriophages or their lysins in fighting against bacterial pathogens [5,12]. In the present study, we studied a *Thermus* phage named TSP4 by sequencing its whole genome, and found that the genome of TSP4 consists of 108 protein-coding genes (GenBank number: MH992131.1; Table S1). Interestingly, these protein-coding genes fall into two major categories: genes in the clockwise direction mainly encode phage structural and morphogenesis proteins, while genes in the counterclockwise direction mainly encode DNA replication, recombination, and nucleotide metabolism-related proteins. This kind of organization in viruses is special and we speculate that it may help the survival of phage TSP4 under extreme conditions, which deserves further study.

Due to the existence of a protective outer membrane (OM) that usually prevents a great number of antibiotics or drugs from entering Gram-negative bacterial cells, there is an increased interest in the identification and characterization of phage lysins that, as an alternative to conventional antibiotics, combat Gram-negative pathogens [29]. However, currently, the number of reported phage lysins that fight against Gram-positive bacteria is well over those of lysins against Gram-negative bacteria [28]. In this study, we found that TSPphg has considerable antimicrobial activity against both Gram-negative and Gram-positive bacteria; to our knowledge, it represents the first such lysin derived from the natural gene sequence in phage genome. Recently, Plotka et al. have reported an endolysin named Ts2631 from the *Thermus scotoductus* phage vB_Tsc2631, which could serve as an agent against alarming MDR Gram-negative bacteria such as MDR clinical strains of *A. baumannii* and *P. aeruginosa* [18]. The ability of Ts2631 to pass bacterial OMs and perform cleavage of the peptidoglycan is mediated by its unique 20-residue N-terminus, which contains seven positively charged amino acids [30]. Very similarly, we found that the 20-residue N-terminal tail of TSPphg (MRLPTKTSRFGYVHGQRNHE) contains six positively charged residues (underlined) as well. Moreover, in silico analysis using SMS v2.0 [31] predicted the isoelectric point of TSPphg to be 9.56. Considering the very short length of the TSPphg protein (166 aa, GenBank number: QAY18185.1), we hypothesize that it may be able to recognize bacteria by electrostatic interactions with negatively charged molecules on the bacterial surface (for example, lipoteichoic acid (LTA) and lipid A in Gram-positive and Gram-negative bacteria, respectively) [32,33,34]. Very likely, this nonreceptor-mediated mechanism could contribute to penetration of TSPphg through the OMs of Gram-negative bacteria [35,36], and thus be responsible for the observed antimicrobial activity of TSPphg lysin. Overall, these clues suggest that TSPphg could be a multifunctional lysin with bacterial OM-interfering and peptidoglycan-degrading activities. Future structure-based functional studies should delineate the molecular mechanism involved more precisely.

In conclusion, by sequencing and annotating the whole genome of phage TSP4, we characterized a new phage lysin named TSPphg. In vitro and in vivo examination further confirmed its bactericidal activity against both Gram-negative and Gram-positive bacteria. Given the well-presented concerns associated with the emergence and persistence of antibiotic-resistant strains of pathogenic bacteria, TSPphg may yet represent a viable phage-derived alternative to antibiotics.

## Figures and Tables

**Table 1:** Bactericidal activity of TSPphg against various strains of Gram-negative or Gram-positive bacteria.

Strain	Antibiotic Resistant ^a^	Starting Bacterial Count (Log_10_ CFU/mL)	Log_10_ of (Starting Count/Final Count) ^b^
*S. paratyphi* B			
CMCC(B)50094	None	7.66 ± 0.09	3.91 ± 0.25
*E. coli* O157			
KUST401	STR, TET and AMP	7.63 ± 0.20	3.03 ± 0.34
*K. pneumoniae*			
14V0622	CRO, AMP, CFZ, ATM, FOX and FEP	8.31 ± 0.10	6.12 ± 0.10
1501SP0122	AMP, CFZ, ATM, AMX and FEP	8.16 ± 0.14	6.41 ± 0.07
13A14918	CRO, AMP, CFZ, NIT, AMP, AMX and GEN	7.48 ± 0.16	> 6.48 (*)
13A15188	AMP and NIT	7.42 ± 0.18	2.45 ± 0.01
13V1837	CRO, AMP, CFZ, ATM, NIT and FEP	8.05 ± 0.04	1.09 ± 0.40
1501SP0351	CRO, AMP, CFZ, ATM, AMX and FEP	8.47 ± 0.01	1.90 ± 0.01
13A14165	AMP, NIT and CFP	8.57 ± 0.05	3.05 ± 0.20
14A0287	CRO, AMP, TZB, CFZ, ATM, NIT, FOX and AMK	7.74 ± 0.03	> 6.74 (*)
1412SP0200	AMP, NIT and CIP	7.82 ± 0.05	> 6.82 (*)
*B. subtilis*			
CMCC(B)63501	None	6.11 ± 0.08	1.99 ± 0.22
*Staphylococcus epidermidis*			
ATCC12228	None	5.57 ± 0.12	2.77 ± 0.26
*Micrococcus luteus*			
ATCC4698	None	7.81 ± 0.09	> 6.81 (*)
*S. aureus*			
1606BL1486	CIP, CLI, ERY, GEN, LVX, LZD, MXF, NIT, OXA, PEN and RIF	6.67 ± 0.06	2.96 ± 0.43
ATCC6538	None	7.76 ± 0.05	2.11 ± 0.12

^a^ STR, streptomycin; TET, tetracycline; AMP, ampicillin; CRO, ceftriaxone; CFZ, cefazolin; ATM, aztreonam; FOX, cefoxitin; FEP, cefepime; CFZ, cefazolin; AMX, amoxicillin; NIT, nitrofurantoin; GEN, gentamicin; CFP, cefoperazone; TZB, tazobactam; AMK, amikacin; CIP, ciprofloxacin; CLI, clindamycin; ERY, erythromycin; LVX, levofloxacin; LZD, linezolid; MXF, moxifloxacin; OXA, oxacillin; PEN, penicillin; RIF, rifampin. ^b^ All values represent mean ± standard deviation. For all tests, the log reduction for TSPphg treatment was measured after incubating different bacterial cells with 50 μg/mL TSPphg dissolved in PBS at 37 °C for 1 h, and three independent replicates were run in each reaction. The asterisks indicate that after TSPphg treatment, the number of viable cells was below the limit of detection (<10 CFU/mL).

## Supplementary Materials

The following are available online at https://www.mdpi.com/1999-4915/12/2/192/s1, Figure S1: Effects of pH values (A) and NaCl concentrations (B) on the lytic activity of TSPphg; Figure S2: Effect of EDTA on the lytic activity of TSPphg; Figure S3: Thermostability of TSPphg. The lysin was first incubated at different temperatures (from 10 to 85 °C) for 30 min, and then its activity was determined by the standard turbidity reduction assay against *Thermus* sp. TC4 cells; Figure S4: Bactericidal activity of TSPphg against different bacteria over a range of concentrations was indicated in terms of log_10_ reduction; Table S1: Genetic features of open reading frames in the *Thermus* phage TSP4 genome.
