# Supplementary material for: TSPphg Lysin from the Extremophilic Thermus Bacteriophage TSP4 as a Potential Antimicrobial Agent against Both Gram-Negative and Gram-Positive Pathogenic Bacteria
Source: Viruses. 2020 Feb 9;12(2):192. doi: 10.3390/v12020192 (PMC7077265; doi:10.3390/v12020192)
Supplement: Supplementary file 1 [file viruses-12-00192-s001.pdf]

This document contains the following supplementary materials:

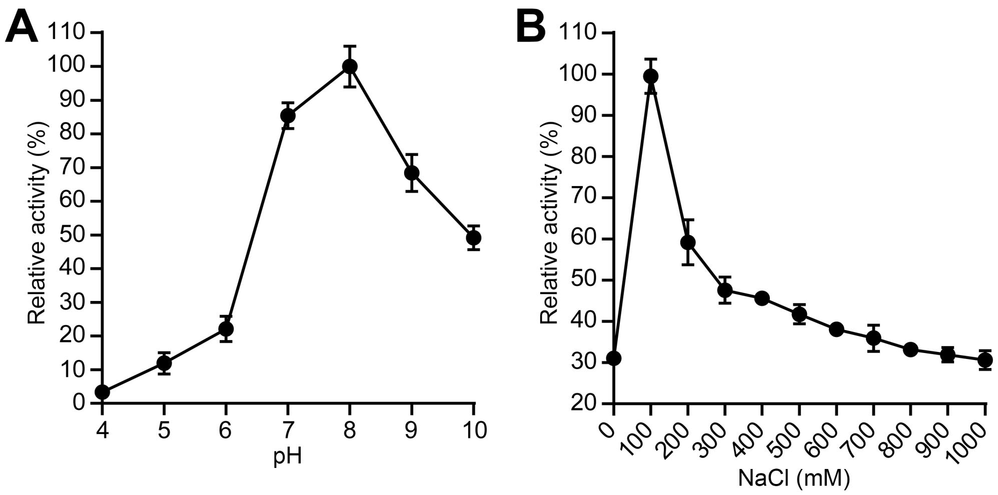

Figure S1. Effects of pH values (A) and NaCl concentrations (B) on the lytic activity of TSPphg. Relative activity of TSPphg against *Thermus* sp. TC4 (the host bacterium for phage TSP4) cells was determined by comparing the lytic activity at specific condition with the maximal activity achieved (= 100%). Each experiment was repeated in triplicate, and error bars represent the standard deviation.

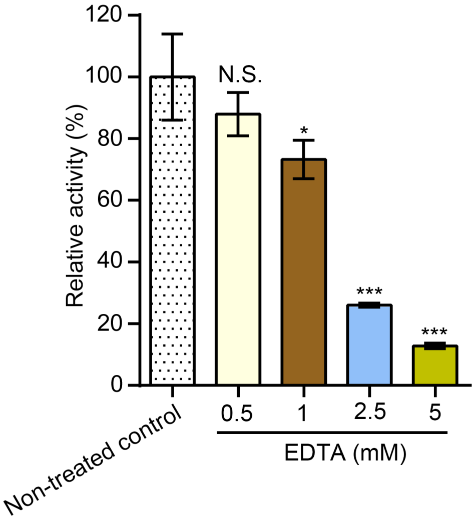

Figure S2. Effect of EDTA on the lytic activity of TSPphg. The activity of TSPphg was analyzed at 60 ˚C for 1 h against *Thermus* sp. TC4 cells in the absence (non-treated control) or in the presence of EDTA (from 0.5 to 5 mM) and indicated as a percentage in relation to the non-treated control. Each experiment was repeated in triplicate, and error bars denote the standard deviation. P values were determined by comparing the lytic activity at specific condition with the non-treated control using Student's t test. N.S., not significant; *, $P < 0.05$; ***, $P < 0.001$.

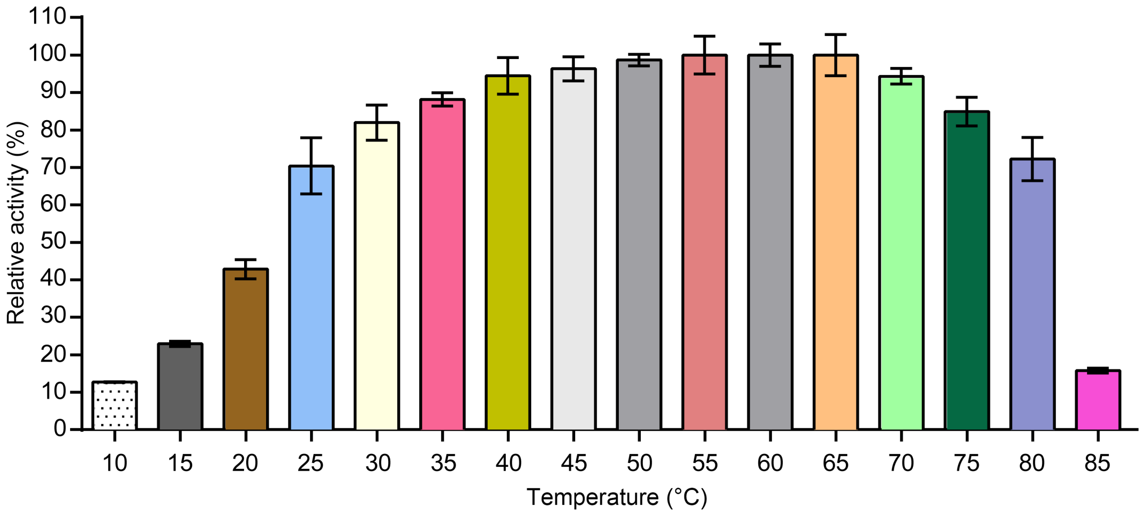

Figure S3. Thermostability of TSPphg. The lysin was first incubated at different temperatures (from 10 to 85 ˚C) for 30 min, and then its activity was determined by the standard turbidity reduction assay against *Thermus* sp. TC4 cells. All data were normalized to the maximal lytic activity among the dataset (= 100%) and presented as mean ± standard deviation of n=3 experiments.

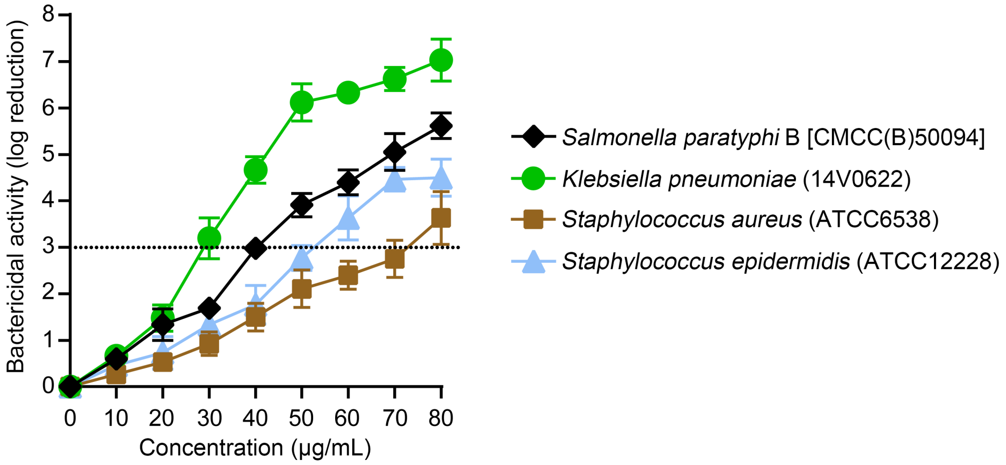

Figure S4. Bactericidal activity of TSPphg against different bacteria over a range of concentrations was indicated in terms of log_{10} reduction. All the bacterial cells were exposed to increasing concentrations of TSPphg in PBS at 37˚C for 1 h, serially diluted and plated for colony counts. Results represent the mean ± standard deviation of n=3 experiments.

Table S1. Genetic features of open reading frames in the *Thermus* phage TSP4 genome.

ORF name	Start	Stop	Strand	Gene length	Description	Best hit NCBI accession [a]	Identity %	E-value
ORF1	1168	65	-	1104	Hypothetical protein	No hit	No hit	No hit
ORF2	1440	1165	-	276	Hypothetical protein	No hit	No hit	No hit
ORF3	1792	1421	-	372	Hypothetical protein	No hit	No hit	No hit
ORF4	2989	1838	-	1152	IS200/IS605 family element transposase accessory protein TnpB	WP_008632327	85.21	0
ORF5	2501	3007	+	507	Hypothetical protein	No hit	No hit	No hit
ORF6	3396	3112	-	285	Helix-turn-helix transcriptional regulator	WP_003049748	43.86	5.01E-06
ORF7	4222	3350	-	873	UvrD/REP helicase	KKP71355	24.91	6.47E-09
ORF8	4638	4267	-	372	Hypothetical protein	No hit	No hit	No hit
ORF9	5168	4698	-	471	Hypothetical protein HRbin39_00114	GBD40745	48.72	1.90E-43
ORF10	5062	5466	+	405	Hypothetical protein	No hit	No hit	No hit
ORF11	7409	5190	-	2220	DNA polymerase I, partial	RIZ70316	31.49	2.23E-73
ORF12	5883	6290	+	408	Hypothetical protein	No hit	No hit	No hit
ORF13	6639	7154	+	516	Hypothetical protein	No hit	No hit	No hit
ORF14	8013	7351	-	663	Hypothetical protein	No hit	No hit	No hit
ORF15	8511	8014	-	498	Hypothetical protein	No hit	No hit	No hit
ORF16	8885	8520	-	366	Hypothetical protein	No hit	No hit	No hit
ORF17	9230	8895	-	336	Hypothetical protein	No hit	No hit	No hit
ORF18	10743	9247	-	1497	Ribonucleoside-triphosphate reductase, adenosylcobalamin-dependent	WP_061429851	48.30	2.50E-87

ORF19	12036	10774	-	1263	Ribonucleotide reductase and Pyruvate formate lyase	WP_133008149	43.06	1.03E-89
ORF20	10895	11395	+	501	Hypothetical protein	No hit	No hit	No hit
ORF21	12946	12044	-	903	FAD-dependent thymidylate synthase	WP_038070007	51.91	2.46E-76
ORF22	13620	13063	-	558	Hypothetical protein, partial	WP_122653417	48.74	7.97E-31
ORF23	14376	13897	-	480	Hypothetical protein	WP_117446303	32.84	5.42E-09
ORF24	14630	14373	-	258	Hypothetical protein	No hit	No hit	No hit
ORF25	15144	14788	-	357	Hypothetical protein	No hit	No hit	No hit
ORF26	15634	15071	-	564	dCTP deaminase	RLE16834	36.31	9.55E-13
ORF27	15983	15723	-	261	Hypothetical protein	No hit	No hit	No hit
ORF28	16560	15955	-	606	Hypothetical protein	No hit	No hit	No hit
ORF29	17184	16702	-	483	Hypothetical protein	No hit	No hit	No hit
ORF30	17727	16795	-	933	Hypothetical protein	No hit	No hit	No hit
ORF31	18332	17829	-	504	Hypothetical protein	No hit	No hit	No hit
ORF32	18756	18355	-	402	Hypothetical protein	No hit	No hit	No hit
ORF33	19191	18811	-	381	Hypothetical protein	No hit	No hit	No hit
ORF34	20267	19251	-	1017	Eukaryotic type DNA primase small subunit	WP_155298980	32.97	7.74E-33
ORF35	20553	20254	-	300	Hypothetical protein	No hit	No hit	No hit
ORF36	20749	20477	-	273	Hypothetical protein	No hit	No hit	No hit
ORF37	21048	20746	-	303	Hypothetical protein	No hit	No hit	No hit
ORF38	21372	21049	-	324	Hypothetical protein	No hit	No hit	No hit
ORF39	21767	21300	-	468	Archaeal-type Holliday junction resolvase	WP_050781077	49.12	1.04E-08

ORF40	21766	22347	+	582	Hypothetical protein	No hit	No hit	No hit
ORF41	22335	21769	-	567	Hypothetical protein	No hit	No hit	No hit
ORF42	23570	22344	-	1227	AAA family ATPase	WP_122653455	25.35	4.49E-21
ORF43	24202	23567	-	636	TPA: hypothetical protein DEG70_08706	HBY46318	33.98	9.92E-21
ORF44	24601	24377	-	225	Hypothetical protein	No hit	No hit	No hit
ORF45	24938	24507	-	432	Hypothetical protein	No hit	No hit	No hit
ORF46	26683	24932	-	1752	Hypothetical protein	No hit	No hit	No hit
ORF47	27306	26692	-	615	Molecular chaperone	KUO70647	34.23	2.58E-10
ORF48	27770	27495	-	276	Hypothetical protein	No hit	No hit	No hit
ORF49	28318	27908	-	411	Hypothetical protein P74p51	WP_119358636	43.28	1.33E-23
ORF50	28595	28356	-	240	Hypothetical protein	No hit	No hit	No hit
ORF51	28938	28600	-	339	Hypothetical protein	No hit	No hit	No hit
ORF52	30003	29035	-	969	Hypothetical protein BGO52_03765	OJW00213	31.50	2.01E-06
ORF53	31283	30348	-	936	Hypothetical protein	WP_062542435	34.29	2.80E-35
ORF54	31904	31287	-	618	Hypothetical protein	No hit	No hit	No hit
ORF55	32752	32186	-	567	Hypothetical protein	No hit	No hit	No hit
ORF56	33287	32805	-	483	Hypothetical protein	No hit	No hit	No hit
ORF57	33810	33343	-	468	Hypothetical protein	No hit	No hit	No hit
ORF58	34139	33852	-	288	Hypothetical protein	No hit	No hit	No hit
ORF59	35216	34341	-	876	Hypothetical protein	No hit	No hit	No hit
ORF60	35203	35574	+	372	Hypothetical protein	No hit	No hit	No hit
ORF61	37534	35627	-	1908	Hypothetical protein	No hit	No hit	No hit

ORF62	36800	37390	+	591	Hypothetical protein	No hit	No hit	No hit
ORF63	38194	37610	-	585	Hypothetical protein AFE_1197	WP_011254754	48.87	1.50E-46
ORF64	39444	38998	-	447	Ribonuclease HI	WP_038070204	82.64	5.21E-85
ORF65	40033	39764	-	270	Hypothetical protein	No hit	No hit	No hit
ORF66	40280	40053	-	228	Hypothetical protein	No hit	No hit	No hit
ORF67	40454	40747	+	294	Hypothetical protein	No hit	No hit	No hit
ORF68	41385	42041	+	657	Hypothetical protein P23p80	WP_122653456	31.12	2.78E-17
ORF69	41998	42210	+	213	Hypothetical protein	No hit	No hit	No hit
ORF70	42212	42931	+	720	ParB-like nuclease domain-containing protein	WP_130123024	38.89	4.24E-07
ORF71	43229	42873	-	357	Hypothetical protein	No hit	No hit	No hit
ORF72	43303	43563	+	261	Hypothetical protein	No hit	No hit	No hit
ORF73	44073	43870	-	204	Hypothetical protein	No hit	No hit	No hit
ORF74	44000	45004	+	1005	Terminase	WP_155299024	42.18	1.15E-65
ORF75	45825	44902	-	924	Hypothetical protein	No hit	No hit	No hit
ORF76	45007	46344	+	1338	Portal protein	GAK61796	27.08	1.00E-31
ORF77	46341	47360	+	1020	Hypothetical protein	No hit	No hit	No hit
ORF78	47372	47815	+	444	Hypothetical protein	No hit	No hit	No hit
ORF79	47826	49055	+	1230	Major head protein	WP_155299028	27.55	1.81E-33
ORF80	49065	49268	+	204	Hypothetical protein	No hit	No hit	No hit
ORF81	49269	49667	+	399	Hypothetical protein	No hit	No hit	No hit
ORF82	49664	50542	+	879	Hypothetical protein	No hit	No hit	No hit
ORF83	50539	51009	+	471	Hypothetical protein	No hit	No hit	No hit
ORF84	51022	52068	+	1047	Hypothetical protein	No hit	No hit	No hit

ORF85	52074	52568	+	495	Hypothetical protein	No hit	No hit	No hit
ORF86	52609	67698	+	15090	Phage tail tape measure protein	WP_035399815	36.13	3.00E-09
ORF87	55067	54591	-	477	Hypothetical protein	No hit	No hit	No hit
ORF88	56145	55606	-	540	Hypothetical protein	No hit	No hit	No hit
ORF89	57333	56497	-	837	Hypothetical protein	No hit	No hit	No hit
ORF90	57806	57315	-	492	Hypothetical protein	No hit	No hit	No hit
ORF91	62114	61593	-	522	Hypothetical protein	No hit	No hit	No hit
ORF92	67769	68347	+	579	Hypothetical protein	No hit	No hit	No hit
ORF93	68344	69030	+	687	Hypothetical protein	No hit	No hit	No hit
ORF94	69030	70157	+	1128	Hypothetical protein	No hit	No hit	No hit
ORF95	70150	73731	+	3582	Hypothetical protein P74p99	WP_155299040	35.51	2.70E-08
ORF96	73731	75377	+	1647	Phage tail-collar fibre protein, partial	SHJ28085	26.98	2.82E-16
ORF97	75390	75761	+	372	Hypothetical protein	No hit	No hit	No hit
ORF98	75804	77387	+	1584	Hypothetical protein	No hit	No hit	No hit
ORF99	77601	78494	+	894	Hypothetical protein GLX28_02960	MXV18596	42.40	4.13E-11
ORF100	78491	78757	+	267	Hypothetical protein	No hit	No hit	No hit
ORF101	78747	79100	+	354	Hypothetical protein	No hit	No hit	No hit
ORF102	79084	79584	+	501	M23 family metallopeptidase	WP_119315009	36.11	1.00E-12
ORF103	79581	79940	+	360	Hypothetical protein	No hit	No hit	No hit
ORF104	79937	80800	+	864	Hypothetical protein	WP_041051483	29.86	1.04E-19
ORF105	80927	81379	+	453	Hypothetical protein	No hit	No hit	No hit
ORF106	81388	81963	+	576	Hypothetical protein	No hit	No hit	No hit
ORF107	81975	82745	+	771	Hypothetical protein	No hit	No hit	No hit

ORF108	82758	83003	+	246	Hypothetical protein	No hit	No hit	No hit

[a] Protein sequences of the predicted ORFs of *Thermus* phage TSP4 were subjected to BLASTP program to analyze their best known matches on the NCBI website (https://blast.ncbi.nlm.nih.gov). The NCBI non-redundant database (nrdb) was used as the reference database, with the cutoff E-value set at 1E-05.
